# Tumor-type agnostic, targeted therapies make a new step forward: The first tumor-agnostic approval of a HER2-targeted therapy

**DOI:** 10.17305/bb.2024.10641

**Published:** 2024-08-01

**Authors:** Semir Vranic, Zoran Gatalica

**Affiliations:** 1 College of Medicine, QU Health, Qatar University, Doha, Qatar; 2 Reference Medicine, Phoenix, AZ, United States of America

Oncologic treatment has recently undergone substantial therapeutic paradigm shifts, from classical tumor-specific and biomarker-agnostic approaches to more molecular, biomarker-specific, and tumor-agnostic. Tumor-type (histology) agnostic drugs work across cancer types and present a novel shift in precision oncology [1]. Compared with traditional cancer therapies, this novel approach implies molecularly informed treatment strategies and enables targeted treatment regardless of tumor histology (type). Such drugs are usually utilized in small clinical cohorts with diverse tumor types sharing a common genomic event (molecular biomarker). One of the key elements of this approach is the presence of a common biomarker across many tumor types. Biomarker predicts response to the targeted drugs, as well as deciphers potential resistance mechanisms.

Since its first approval in May 2017 (pembrolizumab for cancers deficient in mismatch repair [dMMR] or with high microsatellite instability [MSI]), several additional tumor-agnostic drugs have been approved through April 2024. These include neurotrophic tyrosine receptor kinase (NTRK) inhibitors larotrectinib and entrectinib, dorstarlimab-gxly (an anti-PD-1 antibody), pembrolizumab for cancers with high tumor mutational burden (TMB-H), the combination of dabrafenib and trametinib for *BRAF^V600E^* gene mutated cancers, and selpercatinib for *RET*-fused cancers [1].

Human epidermal growth factor receptor 2 (HER2/neu) is a tyrosine kinase receptor whose expression has been described in various cancers, e.g., breast, gastric, and gastroesophageal. In addition, lung, colorectal, biliary tract, bladder, cervical, endometrial, ovarian, prostate, and pancreatic cancers may also overexpress HER2/neu protein in a substantial proportion of cases although routine testing is not performed in these cancers [2–7]. Oncogenic HER2/neu protein activation is usually caused by the *HER2/neu* (*ERBB2*) gene amplification and less commonly due to the *HER2/neu* (*ERBB2*) gene mutations [8–13]; both genomic alterations are associated with aggressive clinical course and poor outcome. At low levels, HER2/neu protein expression (without *HER2/neu* gene alterations) is required for normal cell growth [14].

Anti-HER2 treatment modalities have been used to treat breast, gastric, lung, and colorectal cancers. The anti-HER2 antibody trastuzumab has been a cornerstone and effective therapy in the treatment of HER2-positive breast cancer since 1998. It has also been approved for the treatment of gastric and gastroesophageal junction cancers [15]. However, the number of approved anti-HER2 therapeutics has rapidly evolved, including tyrosine kinase inhibitors (lapatinib, neratinib, and tucatinib), antibodies (pertuzumab), and antibody–drug conjugates (ADCs) (ado-trastuzumab emtansine [T-DM1] and trastuzumab deruxtecan [DS-8201]) [15, 16].

The most recent tumor-agnostic approval (April 2024) comes from AstraZeneca and Daiichi Sankyo’s Enhertu for trastuzumab deruxtecan (DS-8201). This drug has been approved in the United States for the treatment of adult patients with unresectable/metastatic HER2-positive (defined as score 3+ by immunohistochemistry [IHC]) solid cancers who have received prior systemic therapy and have no satisfactory alternative therapeutic options [17]. Trastuzumab deruxtecan (DS-8201) is a specifically designed ADC against HER2/neu. It was initially discovered by Daiichi Sankyo and jointly developed and commercialized by AstraZeneca and Daiichi Sankyo. It is an ADC composed of an anti-HER2 antibody, a cleavable tetrapeptide-based linker, and a cytotoxic drug acting against topoisomerase I (Topo 1). Trastuzumab deruxtecan (DS-8201) has previously been approved for the treatment of advanced/metastatic HER2+ (score 3+) breast cancer, advanced/metastatic HER2-low (defined by scores 1+ or 2+ without *HER2/neu* gene amplification) breast cancer, HER2+ advanced gastric cancer, and HER2-mutated non-small cell lung cancer (NSCLC) [18–21].

The tumor-agnostic approval for trastuzumab deruxtecan (DS-8201) was based on clinically meaningful benefits (objective response rate [ORR] and duration of response [DoR]) reported in three separate phase II clinical trials (DESTINY-PanTumor02, DESTINY-Lung01, and DESTINY-CRC02).

The DESTINY-PanTumor02 phase II trial included 267 pretreated patients with various HER2 expressions (from 0/unknown to 3+ score by IHC) in different cancer subtypes (biliary tract, bladder, cervical, endometrial, ovarian, pancreatic, or other tumors) [22]. In the entire cohort, the ORR was 37.1%, with a median DoR of 11.3 months; the median progression-free survival (PFS) was 6.9 months, while the overall survival (OS) was 13.4 months. However, the highest ORR was observed in patients whose cancers had centrally confirmed HER2 IHC positivity (score 3+), including endometrial (84.6%), cervical (75%), ovarian (63.6%), urothelial and biliary tract (56.3%, respectively), and other cancer subtypes (44.4%). Notably, no response was recorded in two patients with HER2+ pancreatic cancer [22].

In DESTINY-Lung01, 91 patients with HER2-mutated NSCLC were treated with trastuzumab deruxtecan [23]. 86% of samples had exon 20 insertions. HER2/neu protein expression and *HER2/neu* gene amplification status were evaluated in subsets of cases. Any HER2/neu protein expression (scores 1+ to 3+) was detected in 44 of 53 patients (83%), whereas nine patients had no detectable HER2/neu expression (17%). *HER2/neu* gene was amplified in only 2/45 cases (4%). Nevertheless, clinical ORR to trastuzumab deruxtecan was observed in patients with different *HER2/neu* mutations, as well as in patients without detectable HER2/neu protein expression or *HER2/neu* gene amplification. Overall ORR was seen in 55% of the patients, with a median DoR of 9.3 months, median PFS of 8.2 months, and median OS of 17.8 months [23].

DESTINY-CRC02 trial is an international, randomized, two-arm, parallel, multicenter phase II trial that evaluated the efficacy and safety of two different doses (5.4 or 6.4 mg/kg) of trastuzumab deruxtecan. The study enrolled 122 patients with locally advanced, unresectable, or metastatic HER2+ colorectal cancers (score 3+ by IHC or 2+ confirmed positive by in situ hybridization) [24]. Molecularly, colorectal cancer samples had *BRAF* wild type, and either *RAS* wild-type or mutant tumor type. All patients were previously treated with standard treatment modalities, including anti-HER2 therapies. The trial was completed in two stages. In the first stage, 80 patients were randomized 1:1 to receive either 5.4 or 6.4 mg/kg of trastuzumab deruxtecan. In the second stage, an additional cohort of 42 patients was enrolled, receiving only a 5.4 mg/kg dose of trastuzumab deruxtecan [25]. Most patients in both 5.4 and 6.4 mg/kg trastuzumab deruxtecan arms had HER2/neu score 3+ (78% and 85%), *RAS* wild type colorectal carcinoma (82.9% and 85%), and a median of three and four prior lines of therapy, respectively. ORR (all partial responses) was 37.8% (95% CI, 27.3%–49.2%) in the 5.4 mg/kg arm and 27.5% (95% CI, 14.6%–43.9%) in the 6.4 mg/kg arm. The therapeutic efficacy of trastuzumab deruxtecan was observed irrespective of *RAS* gene mutational status at 5.4 mg/kg of trastuzumab deruxtecan, and in those previously treated with anti-HER2 therapies [25].

Taken together, it appears that trastuzumab deruxtecan may exhibit substantial antitumor activity across the histotypes and beyond the previously approved cancers. Although its activity is markedly higher in HER2+ cancers (score 3+ or amplified), it may also be seen in cancers with low or negative HER2/neu protein expression and different *HER2/neu* mutations (e.g., NSCLC). An accurate (central) assessment of HER2/neu status remains a crucial step in the proper management of cancer patients potentially eligible for treatment with trastuzumab deruxtecan. The second target of trastuzumab deruxtecan (payload cytotoxic drug) is Topo 1, which is not a validated predictive biomarker. In addition, biomarkers of resistance to trastuzumab deruxtecan are another important obstacle. Therefore, the development of double biomarker assessment (targeting HER2/neu and Topo 1) along with comprehensive genomic profiling may be reliable alternatives, not only to properly identify predictive biomarkers of response but also resistance biomarkers [26]. Continued (final) approval of trastuzumab deruxtecan in a tumor-agnostic approach will depend upon further verification of its clinical benefits in confirmatory clinical trials.
